# Hepatic Nampt Deficiency Aggravates Dyslipidemia and Fatty Liver in High Fat Diet Fed Mice

**DOI:** 10.3390/cells12040568

**Published:** 2023-02-10

**Authors:** Dao-Xin Wang, Sheng-Li Qing, Zhu-Wei Miao, Heng-Yu Luo, Jia-Sheng Tian, Xiu-Ping Zhang, Shu-Na Wang, Tian-Guang Zhang, Chao-Yu Miao

**Affiliations:** Department of Pharmacology, Second Military Medical University (Naval Medical University), Shanghai 200433, China

**Keywords:** Nampt, dyslipidemia, serum lipids, fatty liver, hepatopathy, hepatocyte

## Abstract

Nicotinamide phosphoribosyltransferase (Nampt) is the rate-limiting enzyme in the salvage pathway of nicotinamide adenine dinucleotide (NAD) biosynthesis. Thus far, hepatic Nampt has not been extensively explored in terms of its effects on serum lipid stability and liver lipids metabolism. In this study, hepatocyte-specific Nampt knockout (HC-Nampt^-/-^) mice were generated by Cre/loxP system. Nampt mRNA expression was reduced in the liver, but not in other tissues, in HC-Nampt^-/-^ mice compared with wild-type (WT) mice. Hepatic Nampt deficiency had no effect on body weight and fasting blood glucose, and it did not induce atherosclerosis in mice under both normal chow diet (NCD) and high fat diet (HFD). At baseline state under NCD, hepatic Nampt deficiency also did not affect liver weight, liver function index, including alanine aminotransferase, aspartate aminotransferase, albumin and alkaline phosphatase, and serum levels of lipids, including triglycerides (TG), total cholesterol (TC), high-density lipoprotein cholesterol (HDL-C), low-density lipoprotein cholesterol (LDL-C), and non-esterified fatty acids (NEFA). However, under HFD, deficiency of hepatic Nampt resulted in increased liver weight, liver function index, and serum levels of TG, TC, HDL-C, and NEFA. Meanwhile, histopathological examination showed increased fat accumulation and fibrosis in the liver of HC-Nampt^-/-^ mice compared with WT mice. Taken together, our results show that hepatic Nampt deficiency aggravates dyslipidemia and liver damage in HFD fed mice. Hepatocyte Nampt can be a protective target against dyslipidemia and fatty liver.

## 1. Introduction

Nicotinamide phosphoribosyltransferase (Nampt), also known as visfatin or pre-B cell colony enhancing factor (PBEF), is a rate-limiting enzyme for biosynthesizing nicotinamide adenine dinucleotide (NAD) in mammals [1,2,3,4]. In addition to the role in energy production, the Nampt-NAD axis connects to sirtuin (SIRT) signaling, constituting a strong endogenous defense system against various stresses [5,6].

Studies in our laboratory on Nampt have shown that ageing mediated NAD pool decrease is caused by decreased Nampt activity, rather than a reduction in the de novo synthesis pathway related enzymes [7]. The Nampt mutant transgenic mice exhibited significantly decreased NAD levels, mediated by a decrease in Nampt activity throughout the body, causing hepatopathy, including inflammation, fibrosis, lipid homeostasis imbalance, and steatosis [7]. However, its effect on serum lipids was not studied. Replenishment with the natural NAD precursor nicotinamide riboside (NR) completely corrected the development of hepatopathy induced by deficiency of NAD. These results suggest that Nampt can protect the liver from metabolic imbalance, and mice with Nampt overexpression can be constructed to explore its therapeutic effect. However, our later study showed that systemic Nampt overexpression aggravated atherosclerosis development in apolipoprotein E knockout (ApoE^-/-^) mice [8]. This study indicates that systemic Nampt activation seems to be a double-edged sword and is not an ideally therapeutic strategy, especially for ageing-related disease, such as fatty liver.

There is widespread expression of Nampt in the tissues, with higher levels of expression found in bone marrow, liver, muscles, and adipose tissue [5,9]. The liver plays an imperative role in maintaining the metabolism of lipids as the main metabolic organ in mammals. Fatty liver refers to an excessive amount of fat store in the liver, which causes the deterioration of hepatic cells [10]. Hepatic lipid metabolism disorders, also known as metabolic fatty liver diseases, are caused by high fat intake and overloaded metabolism [11]. The presence of dyslipidemia (hypercholesterolemia, hypertriglyceridemia, or both) has been reported in part of cases associated with fatty liver [12].

Given that systemic impairment of Nampt-NAD axis results in fatty liver lesions [7], and Nampt knockdown leads to lipid accumulation in HepG2 cells in vitro [13], and we hypothesize that hepatic Nampt plays a critical role in the liver lipid metabolism, and it is sufficient to affect the levels of lipids in liver and blood. In this study, hepatocyte-specific Nampt knockout mice were constructed to investigate the role of hepatic Nampt in the regulation of dyslipidemia and fatty liver, providing novel insight for design of future targeting therapy.

## 2. Materials and Methods

### 2.1. Generation of Hepatocyte-Specific Nampt Knockout Mice

**Figure 1:**
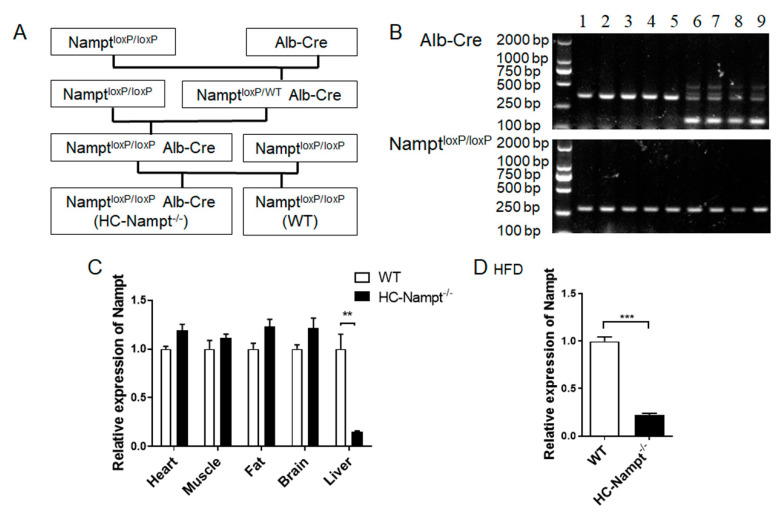
Generating hepatocyte-specific Nampt knockout (HC-Nampt^-/-^) mice. (**A**) Breeding strategy for generating HC-Nampt^-/-^ mice. (**B**) The genotypes of HC-Nampt^-/-^ and WT mice. Lanes 1–5: Nampt^loxP/loxP^, also represent as WT. Lanes 6–9: Nampt^loxP/loxP^ Alb-Cre, also represent as HC-Nampt^-/-^. (**C**) Nampt mRNA expression was detected by real-time PCR in various tissues of adult mice fed NCD. *n* = 5 (WT) or 4 (HC-Nampt^-/-^). (**D**) Nampt mRNA expression was detected by real-time PCR in liver of the mice fed HFD for 10 weeks. *n* = 6 (WT) or 8 (HC-Nampt^-/-^). Data are expressed as the mean ± SEM. Relative levels of HC-Nampt^-/-^ mRNA were normalized to WT, respectively. ** *p* < 0.01 and *** *p* < 0.001 between two groups by two-tailed student’s *t* test.

Nampt^loxP/loxP^ mice and Alb-Cre mice were used to generate a hepatocyte-specific Nampt knockout (HC-Nampt^-/-^) animal model. Alb-Cre transgenic mice were purchased from Shanghai Biomodel Organism Science & Technology Development Co.,Ltd. (Shanghai, China). Cre is a P1 phage-derived site-specific DNA recombinase [14]. It involves identifying and splicing the DNA sequence between two loxP sites, resulting in a single loxP site on a linear DNA molecule when two loxP sites are aligned in the same direction. Albumin (Alb) is specifically and abundantly expressed in the hepatocyte, and as a liver-targeted promoter, it has been widely used to prepare hepatocyte-specific gene knockout mice models [15]. Nampt^loxP/loxP^ mice were initially constructed by Dr. Oberdan Leo and donated to our laboratory. The breeding strategy (Figure 1A) was that Nampt^loxP/loxP^ mice were crossed with Alb-Cre mice to generate Nampt^loxP/WT^Alb-Cre mice, which were crossed with Nampt^loxP/loxP^ mice to generate Nampt^loxP/loxP^Alb-Cre mice. Nampt^loxP/loxP^Alb-Cre mice crossed with Nampt^loxP/loxP^, as maternal generation mice only for reproduction, finally generating offspring, Nampt^loxP/loxP^ Alb-Cre mice, which were HC-Nampt^-/-^ and littermate controls. Nampt^loxP/loxP^ is considered wild type (WT). All Nampt^loxP/loxP^ mice used to breed were homozygous and had no Alb-Cre.

### 2.2. Animal Treatment and Sample Collection

Mice were housed in a standard animal room with the appropriate temperature (23~25 °C), 40–70% humidity, and a 12 h/12 h dark/light cycle condition. During the feeding period, mice were provided with adequate water and normal chow diet (NCD) or high-fat diet (HFD). HFD contained approximately 42.5% fat, 1.3% cholesterol, and 0.3% cholate (Shanghai Slac Laboratory Animal Co. Ltd., Shanghai, China). Mice were fed HFD for 10 weeks.

At the end of the experiments, mice fasted for 12 h (20:00 p.m.–8:00 a.m.) and were sacrificed by an overdose of pentobarbital sodium (100 mg·kg^−1^, i.p., Bio-Light, Shanghai, China). The thoracic cavity was opened, and venous blood (0.6–0.8 mL) was collected from the inferior vena cava. A surgical procedure was performed to expose the abdominal cavity and to remove the liver. The liver weight was recorded after it had been rinsed with cold saline, and the liver weight to body weight ratio was calculated. For histological examination, the left lobe of the liver and the aortic portion of the heart, which cut along the horizontal section of the mitral valve, were fixed with 4% paraformaldehyde. About 200 mg carefully excised tissue samples, including the brain, muscle, fat, and the rest of the liver and heart, were directly ground into small tissue mass for RNA extraction or immediately placed into liquid nitrogen for other experiments.

### 2.3. Genomic DNA Extraction and Genotyping by Polymerase Chain Reaction

The genomic DNA of mice was extracted using a Mouse Tail Genomic DNA kit (CoWin Biosciences, Beijing, China) according to the manufacturer’s instructions. A buffer system was used to bind DNA to the silicon matrix adsorption column. Other substances can be washed and removed using organic solvent. Purified DNA was obtained by eluting with water. In a final reaction mixture consisting of 20 μL, 2 μL of genomic DNA was added with corresponding primers in order to amplify the target gene by polymerase chain reaction (PCR). The amplified products were separated by electrophoresis in 2% agarose gel and analyzed by the bio-electrophoresis image analysis system (Furi Technology Co., Ltd. Shanghai, China), photographed under 312 nm ultraviolet light. Cre primer sequences are as follows: wild-type forward 5′-TGCAAACATCACATGCACAC-3′, common reverse 5′-TTGGCCCCTTACCATAACTG -3′, and mutant forward 5′-GAAGCAGAAGCTTAGGAAGATGG-3′ (yielding the expected PCR product of a 351-bp band with or without additional a 150-bp band). Floxed (loxP-flanked) Nampt allele primer sequences were as follows: forward 5′-TCTGGCTCTGTGTACTGCTGA-3′ and reverse 5′-TTCCAGGCTATTCTGTTCCAG-3′ (yielding the expected PCR product of a 300-bp band).

### 2.4. Serum Lipids Assys

AS described in our previous study [16], the venous blood was placed at room temperature for three hours, then it was centrifuged by 3000× *g* for 20 min at 4 °C to obtain serum samples. The serum samples were sent to Servicebio (Wuhan, China). A biochemical analyzer (Chemray 240, Rayto Life and Analytical Sciences Co., Ltd. Shenzhen, China) with the corresponding reagent (Huili Biology, Changchun, China) was used to measure the serum triglyceride (TG), total cholesterol (TC), high-density lipoprotein cholesterol (HDL-C), and low-density lipoprotein cholesterol (LDL-C).

In the presence of cholesterol hydrolase, cholesterol is hydrolyzed into fatty acids, which are then further oxidized to produce hydrogen peroxide. The Trinder reaction was used to detect hydrogen peroxide concentrations. Calculated using the formula, the TC concentration was determined by comparing the hydrogen peroxide concentration in the cholesterol standard solution to the hydrogen peroxide concentration in the serum. To remove free glycerol from the serum, glycerol kinase was applied. Hydrogen peroxide was produced by breaking down TG with lipoprotein lipase. Follow-up tests were consistent with cholesterol testing methods. Phosphotungstic acid-magnesium reagents were needed to precipitate apoB-containing lipoproteins, and HDL-C was measured from the supernatant. The detection principle was the same as cholesterol. Sulfated polyethylene was used to precipitate and disperse LDL-C, then it was calculated by subtracting the cholesterol in the supernatant without LDL-C from the total cholesterol.

Serum NEFAs were determined using commercial assay kits according to the instructions (Nanjing Jiancheng Bioengineering Institute, Nanjing, China). Acetyl-CoA synthase catalyzed the production of hydrogen peroxide from NEFA. It was consistent with the above method of detecting hydrogen peroxide. All the detection methods were recommended by the Chinese Medical Association.

### 2.5. RNA Isolation and Real-Time PCR Analysis

Real-time PCR was performed as reported in our previous study [17]. Briefly, RNA was separated with TRIzol reagent (Invitrogen, Grand Island, NY, USA) according to the instructions of the manufacturer. Two point five micrograms of RNA were reverse transcribed into cDNA using the M-MLV enzyme (Accurate Biology, Changsha, China). Real-time PCR was performed by using a LightCycler96 Real-Time PCR System (Roche, Mannheim, Germany) with a 2 μL cDNA template added into a 20 μL final reaction mixture. The procedure began with 95 °C for 15 min, followed by 40 cycles of ordinal condition 95 °C for 15 s, 60 °C for 30 s and 72 °C for 30 s. Melt curves were made after each run, and dissociation curves were analyzed to confirm the absence of non-specific PCR products. An analysis of gene expression values was performed using the comparative cycle threshold (CT) method (2^−ΔΔCT^). The relative expression of Nampt was normalized to the level of glyceraldehyde 3-phosphate dehydrogenase (GAPDH). The primers used in real-time PCR are obtained from the NCBI, and their sequences are listed in Table 1.

### 2.6. Atherosclerotic Lesion Analysis

As described in our previous study [8], after perfusion and being fully exposed, the entire aorta and heart were collected by careful removing of kidneys and perivascular adipose tissue under a microscope. For the staining of whole aortic lesions with oil red O (ORO), the aorta was placed in phosphate buffered saline, pinned flat and straightened. In the subsequent step, phosphate buffered saline was replaced with ORO working solution (Sigma-Aldrich). Initially, the aorta was immersed in ORO solution for 15 min, followed by 10 min of incubation with 70% ethanol. Finally, the aorta was washed with double-distilled water. The aorta was photographed with a Nikon camera, and the areas of atherosclerotic plaque were compared with each other.

### 2.7. Histopathological Analysis and Oil Red O Staining

The liver and the aortic root were fixed in 4% paraformaldehyde for 24 h. Then, tissues were embedded in paraffin and cut into serial aortic root cross-sections and liver sections (4 µm) for staining with hematoxylin and eosin (HE; Beyotime Biotechnology, Nantong, China) or Masson’s trichrome, which was only performed in the liver. For ORO staining, tissues were embedded in optimal cutting temperature compound (OCT) tissue freezing medium (Servicebio, Wuhan, China) for obtaining serial aortic root cross-sections and liver frozen sections (8 µm) by freezing microtome (Leica, Germany). A summary of the staining process would be dye immersion dyeing, decolorizing with 75% ethanol or absolute ethanol. Glycerol was then used to cover the section after excess water had been removed. The picture was taken by using an upright optical microscope (Leica Microsystems, Wetzlar, Germany) and quantified by the percentage of red area (ORO staining) or blue area (Masson’s staining) in the overall area with an image analysis system (Image J program, Bethesda, MD, USA).

### 2.8. Measurement of Fasting Blood Glucose

Fasting blood glucose was measured in accordance with our previous report [17]. Briefly, the mice fasted for 12 h (20:00 p.m.–8:00 a.m.) and tail blood glucose was measured by blood glucose instrument (Sinocare, Changsha, China). About one drop of blood was removed from the mouse tail and covered the instrument detection area. The weak electricity was detected by the electrons released from oxidation of glucose to gluconic acid catalyzed by potassium ferricyanide. The blood glucose concentration was obtained by conversion according to the standard concentration.

### 2.9. Serum Biochemical Analysis

After the lipid measurement, the remaining serum samples were packed with dry ice and sent to the Testing & Analysis Center (Naval Medical University) for biochemical analysis with an automatic biochemistry analyzer (Hitachi 7180, Tokyo, Japan) to determine the liver function index including alanine aminotransferase (ALT), aspartate aminotransferase (AST), albumin (ALB), and alkaline phosphatase (ALP).

### 2.10. Statistical Analysis

All results are expressed as means ± SEM. Statistical analysis was performed with two-tailed Student’s *t*-test. Statistical analysis was performed using GraphPad Prism 8 software (GraphPad Software Inc., La Jolla, CA, USA). *p* value ≤ 0.05 was considered statistically significant for differences between two groups.

## 3. Results

### 3.1. Identification of the Hepatocyte-Specific Nampt Knockout Mice

HC-Nampt^-/-^ mice were generated by the breeding strategy, as shown in Figure 1A. A sufficient number of offspring mice were obtained and used in experiments. PCR and agarose gel electrophoresis were used to identify the genotype of randomly selected maternal gene knockout mice and WT mice. All mice should have the 300-bp band represented Nampt^loxP/loxP^. Only a 351-bp band appeared in lanes 1–5 (Figure 1B), indicating no activity of the Cre enzyme (WT). Additional 150-bp band represented positive Cre enzyme in lanes 6–9 (HC-Nampt^-/-^) (Figure 1B). Only mice-identified genotypes can be used to propagate, and offspring mice were followed the same procedure for identification of the genotypes.

The mice were selected to verify the efficiency of gene knockout by real-time PCR. The results showed that Nampt mRNA level in HC-Nampt^-/-^ mice liver was decreased by 85% compared with WT group with statistical significance. Among other detected tissues, including heart, muscle, fat, and brain obtained from the same mice, Nampt mRNA levels did not change between WT and HC-Nampt^-/-^ groups (Figure 1C). The mice fed with HFD were also verified the efficiency of gene knockout by real-time PCR. Consistently, Nampt mRNA level in HC-Nampt^-/-^ mice liver was decreased by 78% compared with WT group after 10-week HFD. These results suggest that hepatocyte-specific Nampt knockout (HC-Nampt^-/-^) model mice were successfully constructed.

### 3.2. Deficiency of Hepatic Nampt Has No Effect on Body Weight, Fasting Blood Glucose, and Serum Lipids Levels under Normal Chow Diet

**Figure 2:**
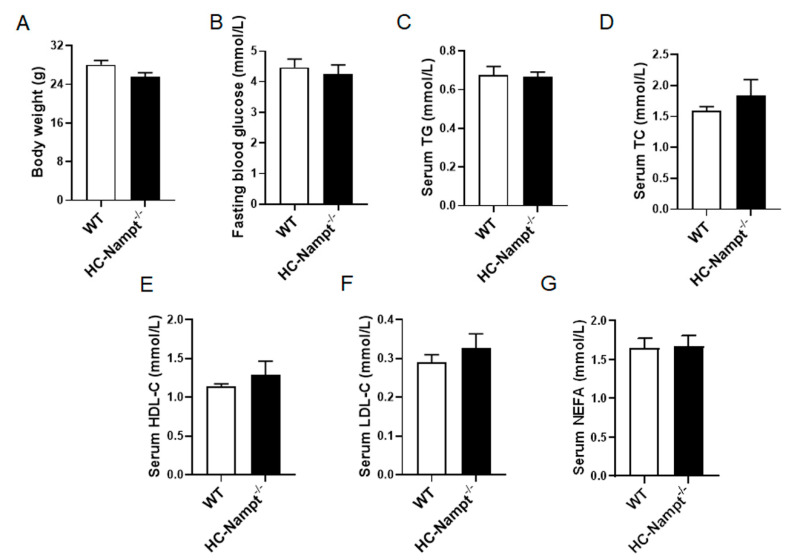
The effects of hepatic Nampt deficiency on body weight, fasting blood glucose, and serum lipids levels in NCD-fed mice. (**A**) Body weight of HC-Nampt^-/-^ and WT mice. (**B**) Fasting blood glucose of HC-Nampt^-/-^ and WT mice. (**C**–**G**) Serum lipids levels of HC-Nampt^-/-^ and WT mice. TG, triglycerides; TC, total cholesterol; HDL-C, high-density lipoprotein cholesterol; LDL-C, low-density lipoprotein cholesterol; NEFA, non-esterified fatty acids. Data are expressed as the mean ± SEM. *n* = 7. No statistical significance between two groups by two-tailed Student’s *t*-test.

To explore the effects of hepatic Nampt deficiency on the physiological indexes under normal condition, we used the NCD-fed mice at the age of 12 months. The body weight was comparable between HC-Nampt^-/-^ and WT mice (Figure 2A). The fasting blood glucose of WT and HC-Nampt^-/-^ mice was found to be within the normal range and not affected by hepatic Nampt deficiency (Figure 2B). Further, the detection of serum lipid parameters showed that serum TG and TC, the main indexes of dyslipidemia and fatty liver, did not change in HC-Nampt^-/-^ mice (Figure 2C,D). Deficiency of hepatic Nampt did not affect HDL-C (Figure 2E). LDL-C was also not altered between the two groups of mice (Figure 2F). NEFA was unchanged either (Figure 2G). The results suggest that the hepatic Nampt deficiency does not affect the body weight and these biochemical parameters at baseline state.

### 3.3. Deficiency of Hepatic Nampt Does Not Alter Liver Weight and Induce Atherosclerosis in Mice under Normal Chow Diet

**Figure 3:**
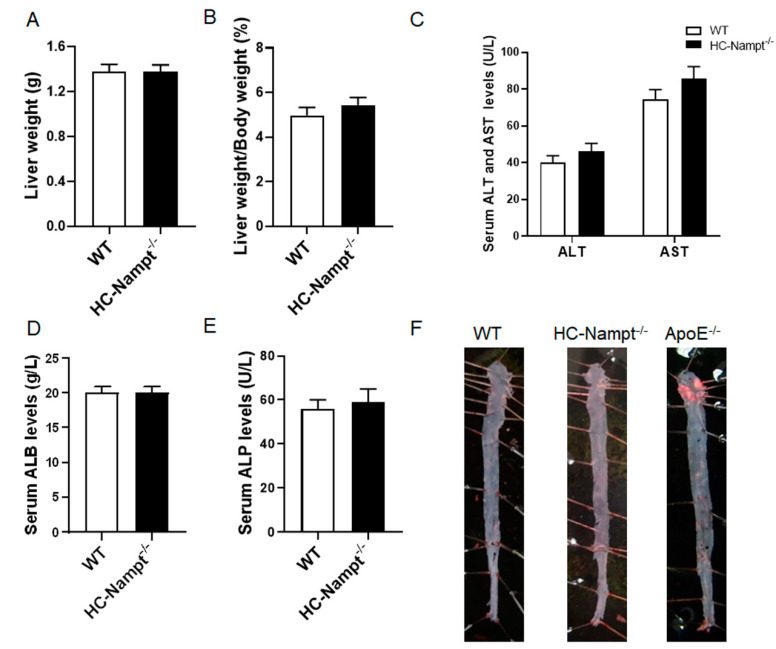
The effects of hepatic Nampt deficiency on liver weight, liver function, and atherosclerotic plaque formation in NCD-fed mice. (**A**,**B**) Liver weight (**A**) and the liver index, i.e., the ratio of the liver weight to the body weight (**B**) in HC-Nampt^-/-^ and WT mice. (**C**–**E**) Serum liver functional indexes of HC-Nampt^-/-^ and WT mice. ALT, alanine aminotransferase; AST, aspartate aminotransferase; ALB, albumin; ALP, alkaline phosphatase. (**F**) Representative images of oil red O staining on the aortas of HC-Nampt^-/-^ and WT mice, as well as ApoE^-/-^ mice. Data are expressed as the mean ± SEM. *n* = 7. No statistical significance was observed between two groups by two-tailed Student’s *t*-test.

The liver as an important metabolically active organ plays a pivotal role in regulating lipid metabolism. The aorta is vulnerable to lipids metabolic abnormalities. Considering these factors, liver and aorta were measured to explore the role of hepatic Nampt in them. In consistent with the above unchanged biochemical parameters, liver weight and liver index (the ratio of liver weight to body weight) were comparable between HC-Nampt^-/-^ and WT mice (Figure 3A,B). There were no changes in liver gross phenotypes between WT and HC-Nampt^-/-^ mice at baseline state (Supplementary Figures S1 and S2, and Table S1). Serum ALT and AST, which are enzymes released primarily by hepatocytes and increased in liver dysfunction, did not differ between the two groups of mice (Figure 3C). ALB is a specific protein synthesized by the hepatocytes, which can effectively evaluate the liver protein synthesis function. There was no difference in serum ALB level between HC-Nampt^-/-^ and WT mice (Figure 3D). When liver-related diseases, such as hepatitis and cirrhosis occur, ALP is overproduced by the hepatocytes and enters the blood through the lymphatic channels. There were no liver lesions observed, as indicated by the unchanged serum ALP level (Figure 3E). There was no obvious oil red O staining in the aortas of both HC-Nampt^-/-^ and WT mice, indicating no lipid deposits, i.e., no atherosclerotic plaque formation, in these two groups of the mice (Figure 3F). In contrast, the aortas of the ApoE^-/-^ mice, an accepted animal model of atherosclerosis as a positive control, displayed the obvious atherosclerotic plaques formation (Figure 3F). These results suggest that, in line with the result of major biochemical parameters, liver and aorta are unaffected by the deficiency of hepatic Nampt at baseline state.

### 3.4. Deficiency of Hepatic Nampt Increases Serum Lipids Levels but Does Not Affect Body Weight and Fasting Blood Glucose in Mice Fed a High-Fat Diet

**Figure 4:**
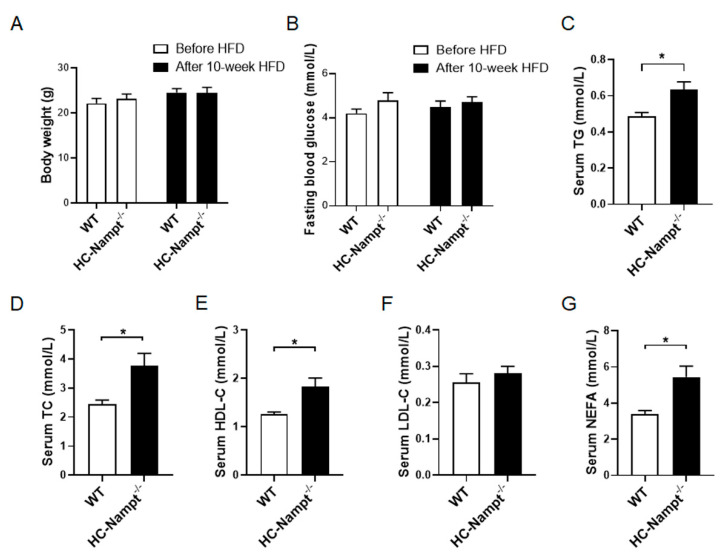
The effects of hepatic Nampt deficiency on body weight, fasting blood glucose, and serum lipids levels in HFD-fed mice. (**A**) Body weight of HC-Nampt^-/-^ and WT mice before and after HFD. (**B**) Fasting blood glucose of HC-Nampt^-/-^ and WT mice before and after HFD. (**C**–**G**) Serum lipids levels of HC-Nampt^-/-^ and WT mice after 10 weeks of HFD. TG, triglycerides; TC, total cholesterol; HDL-C, high-density lipoprotein cholesterol; LDL-C, low-density lipoprotein cholesterol; NEFA, non-esterified fatty acids. Data are expressed as the mean ± SEM. *n* = 6 (WT) or 8 (HC-Nampt^-/-^). * *p* < 0.05 between two groups by two-tailed Student’s *t*-test.

The above results indicate that hepatic Nampt does not affect all examined physiological and biochemical indexes at the baseline state. We used HFD to challenge the mice. Body weight and fasting blood glucose were unchanged before and after 10 weeks of HFD (Figure 4A,B). However, the biochemical analysis showed that serum TG significantly increased by 30% and serum TC by 53% (Figure 4C,D). Serum HDL-C also significantly increased by 45% (Figure 4E), but serum LDL-C only had an increase trend of 10% without statistical significance in HC-Nampt^-/-^ mice compared with WT mice (Figure 4F). Consistent with the change in serum TG, serum NEFA significantly increased by 61% (Figure 4G). These results indicate that hepatic Nampt deficiency aggravates dyslipidemia under HFD challenge.

### 3.5. Deficiency of Hepatic Nampt Increases Liver Weight but Has No Atherosclerosis in Mice Fed a High-Fat Diet

**Figure 5:**
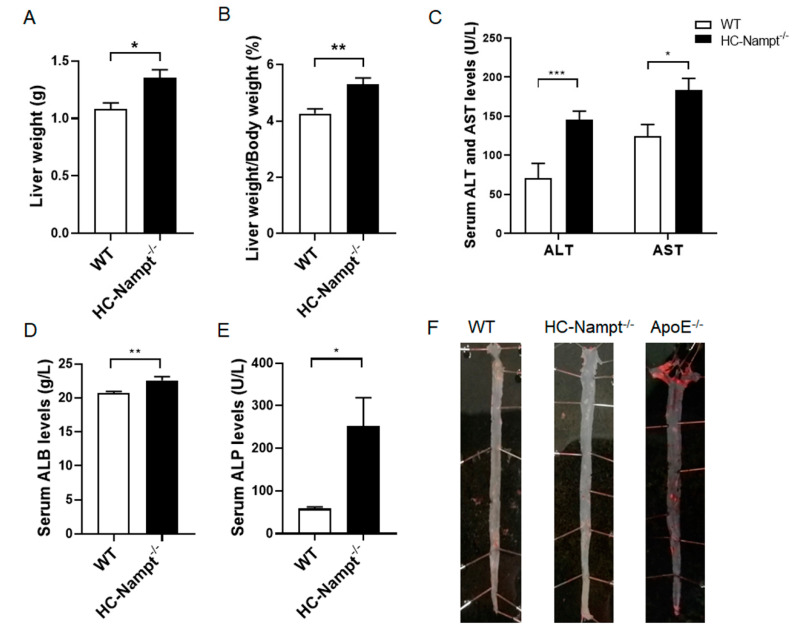
The effects of hepatic Nampt deficiency on liver weight, liver function, and atherosclerotic plaque formation in mice fed a HFD. (**A**,**B**) Liver weight (**A**) and the liver index, i.e., the ratio of the liver weight to the body weight (**B**) in HC-Nampt^-/-^ and WT mice after 10 weeks of HFD. (**C**–**E**) Serum liver functional indexes of HC-Nampt^-/-^ and WT mice after 10 weeks of HFD. ALT, alanine aminotransferase; AST, aspartate aminotransferase; ALB, albumin; ALP, alkaline phosphatase. (**F**) Representative images of oil red O staining on the aortas of HC-Nampt^-/-^ and WT mice after 10 weeks of HFD. The ApoE^-/-^ mice aorta used as a positive control. Data are expressed as the mean ± SEM. *n* = 6 (WT) or 8 (HC-Nampt^-/-^). * *p* < 0.05 and ** *p* < 0.01 between two groups by two-tailed Student’s *t*-test.

In the HFD condition, liver weight increased by 25% in HC-Nampt^-/-^ mice compared with WT mice, suggesting more lipid deposition in the liver of HC-Nampt^-/-^ mice (Figure 5A). Similarly, the liver index significantly increased by 24% in HC-Nampt^-/-^ mice compared with WT mice (Figure 5B). There were alterations in liver gross phenotypes between WT and HC-Nampt^-/-^ mice under HFD challenge (Supplementary Figures S1 and S2, and Table S1). The liver functional indexes were also significantly altered. According to the results, the levels of serum ALT in HC-Nampt^-/-^ mice were twice as high as those in WT mice, and the levels of serum AST were significantly higher by 47% in HC-Nampt^-/-^mice (Figure 5C). ALB increased by only 9%, but the difference was statistically significant between the two groups of mice, suggesting that liver protein synthesis has been mildly affected (Figure 5D). It was observed that the serum ALP levels in HC-Nampt^-/-^ mice were four times larger than those in WT mice, suggesting a severe inflammation in HC-Nampt^-/-^ mice (Figure 5E). Although HFD induced dyslipidemia in hepatic Nampt deficient mice, oil red O staining showed no lipid deposition, i.e., no atherosclerotic plaque formation, in the aortas of both HC-Nampt^-/-^ and WT mice (Figure 5F), which is totally different with the positive atherosclerotic plaque controls in the aortas of ApoE^-/-^ mice.

### 3.6. Deficiency of Hepatic Nampt Induces Liver Histopathological Changes in Mice Fed a High-Fat Diet

**Figure 6:**
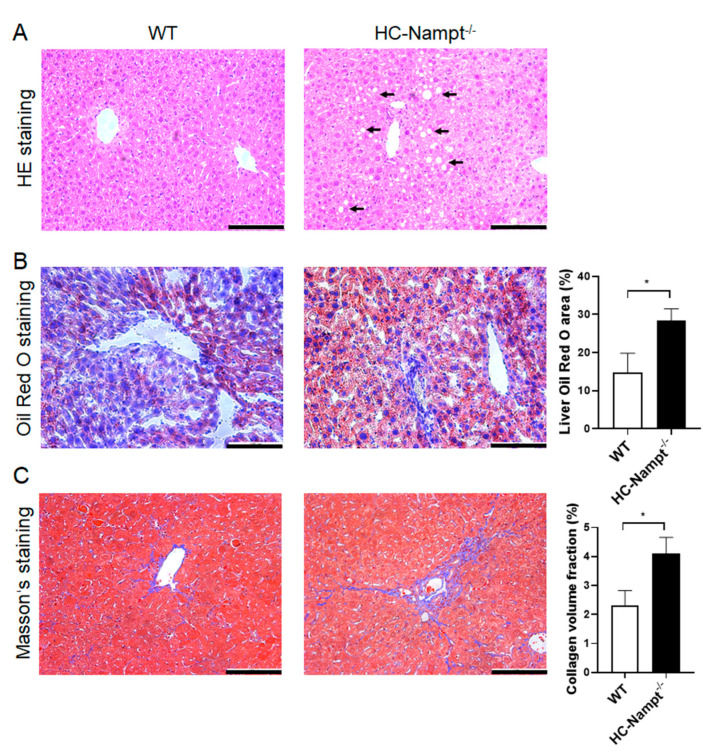
The effect of hepatic Nampt deficiency on liver histology in mice fed a HFD. (**A**) Representative images of HE staining on the liver of HC-Nampt^-/-^ and WT mice after 10 weeks of HFD. (**B**) Representative images and quantitative analysis of oil red O staining on the liver of HC-Nampt^-/-^ and WT mice after 10 weeks of HFD. (**C**) Representative images and quantitative analysis of Masson’s staining on the liver of HC-Nampt^-/-^ and WT mice after 10 weeks of HFD. The scale bar = 100 µm. Data are expressed as the mean ± SEM. *n* = 6 (WT) or 8 (HC-Nampt^-/-^). In quantitative analysis, at least six visual fields were selected for analysis in each sample, and the average value was taken as the data of this sample. * *p* < 0.05 between two groups by two-tailed Student’s *t*-test.

Considering that liver is the main metabolic organ in the organism and the results showed that liver weight was increased by hepatic Nampt deficiency under HFD, we speculated that the liver Nampt deficiency may contribute to fatty liver. HE staining showed that the structure of liver tissue was clear, and the cells were arranged neatly in WT mice fed HFD. On the contrary, the hepatocyte adjacent to the hepatic sinusoids in HC-Nampt^-/-^ mice fed HFD exhibited irregular arrangement and balloon-like changes (Figure 6A). Oil red O staining showed a small amount of lipid deposition in the liver cells of WT mice. However, deficiency of hepatic Nampt aggravated lipid deposition. Quantification by analyzing the proportion of red areas in the whole picture showed that the percentage of oil red O staining area of the HC-Nampt^-/-^ liver was about twice of the WT group (Figure 6B). Masson’s staining can stain collagen fibers blue and other tissues red. According to the staining results, only a small part of lesions in the hepatic sinusoids showed fibrosis in WT mice. In the HC-Nampt^-/-^ mice, the fibrosis area in the hepatic sinusoids was thickened and extended to liver tissue, and the quantitative analysis found that the proportion of blue areas increased by 78% compared with WT mice (Figure 6C). Altogether, these results suggest that hepatic Nampt deficiency results in histopathological damage of the liver in the condition of HFD.

### 3.7. Deficiency of Hepatic Nampt Does Not Change the Aortic Root Morphology in Mice Fed a High-Fat Diet

**Figure 7:**
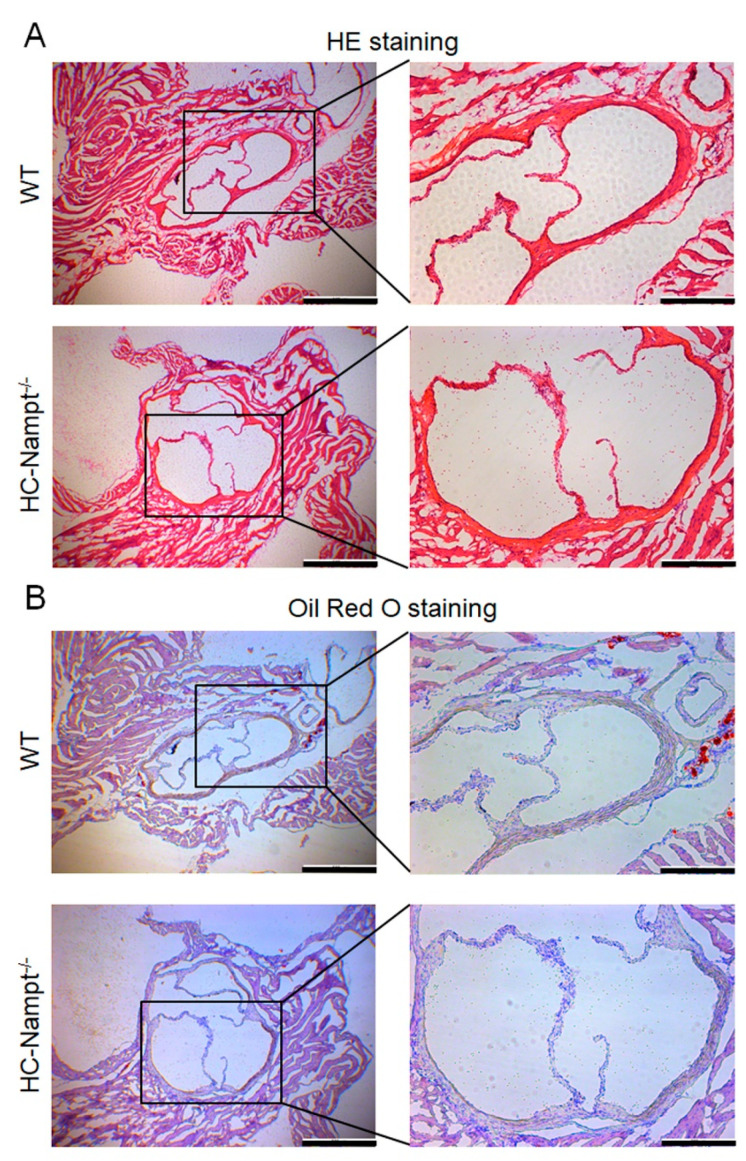
The effect of hepatic Nampt deficiency on aortic root morphology in mice fed a HFD. (**A**) Representative image of HE staining on the aortic root of HC-Nampt^-/-^ and WT mice after 10 weeks of HFD. Global structure shows in the left part, and the scale bar is 500 µm. Local structure shows in the right part, and the scale bar is 200 µm. (**B**) Representative image of oil red O staining on the aortic root of HC-Nampt^-/-^ and WT mice after 10 weeks of HFD. Global structure shows in the left part, and the scale bar is 500 µm. Local structure shows in the right part, and the scale bar is 200 µm.

The aforementioned results indicate that hepatic Nampt deficiency does not induce lipid deposition in the aorta. The aortic root is more prone to lipid deposition and damage than the whole aorta, therefore we detected the aortic root. The HE staining showed that the vascular wall cells in the aortic root were closely arranged, and the aortic valves were intact without injury (Figure 7A). Similarly, oil red O staining showed no lipid deposition in the aortic vessel wall and valves both globally and locally (Figure 7B). 

## 4. Discussion

Our data provide evidence that the deficiency of hepatic Nampt is sufficient to aggravate HFD-induced fatty liver and dyslipidemia. Previous studies have shown that Nampt exerts liver protection by reducing oxidative stress and key necrosis driver gene expression in acetaminophen-induced acute-liver injury [18]. Our previous research also indicates that systemic replenishment with the natural NAD precursor NR completely corrects the development of hepatic steatosis and fatty liver, which is induced by Nampt-mediated NAD deficiency [7]. Although both our laboratory and others have discovered that Nampt-NAD axis can produce beneficial responses in liver [7,19,20,21], the limitation is that these studies are all based on systemic effects. Additionally, we have found that overexpression of systemic Nampt aggravates atherosclerosis in ApoE^-/-^ mice [8], and others have demonstrated that inhibition of Nampt reduces atherosclerosis [22,23]. These studies indicate harmful effects of systemic Nampt activation on atherosclerosis. Considering all these factors, the present study constructed hepatocyte-specific Nampt knockout mice to investigate the role of hepatic Nampt in serum lipids and hepatic steatosis.

Recent studies have shown that HC-Nampt^-/-^ mice constructed using a similar strategy display low hepatic NAD levels and fatty liver [24,25], which are largely consistent with our data that liver injury is aggravated in HC-Nampt^-/-^ mice induced by HFD. They also demonstrate that hepatic Nampt deficiency decreases NAD-related mitochondrial proteins and oxidoreductases that triggers reduced hepatic respiratory capacity. Previously, we also used Nampt inhibitor FK866. Unlike the hepatocyte-specific Nampt knockout that affected only hepatic Nampt, FK866 inhibited Nampt activity throughout the body. FK866 weakened the calorie restriction induced beneficial effects on oxidative stress, mitochondrial biogenesis, and metabolic adaptation [4]. Consistent with our results, inhibition of Nampt by FK866 aggravated hepatic ischemia-reperfusion induced liver damage [26]. FK866 also significantly promoted liver steatosis in the mice fed with HFD [27].

Impaired liver lipid metabolism caused by hepatic Nampt deficiency is associated with NAD-dependent SIRT1 deacetylase, which plays an important role in a variety of pathological and physiological function [28]. Inhibition of Nampt aggravated HFD-induced hepatic steatosis [27], lipid accumulation, and oxidative stress [29] in mice through regulating SIRT1 signaling pathway. Genistein, a primary phytoestrogen in soybean, reduced fat accumulation in chicken hepatocytes by activating the ERβ/FOXO1/Nampt/SIRT1/AMPK signaling pathway [30]. Peripuberty stressed mice showed decreased extracellular Nampt levels and an impaired NAD/SIRT1 pathway in the nucleus accumbens. Normalization of extracellular Nampt levels reduced stress-induced adiposity and reverted alterations in brain function and behavior induced by peripubertal stress through the activation of the NAD/SIRT1 pathway in the nucleus accumbens [31]. These studies suggest that hepatic Nampt deficiency induced Nampt/NAD/SIRT1 pathway inactivation is a critical mechanism for HFD-induced hepatic steatosis and abnormal liver lipid metabolism. However, according to our previous study demonstrating impairment of Nampt-NAD axis in aging and associated fatty liver, oral administration of nicotinamide riboside, a natural NAD precursor, completely corrected the fatty liver phenotypes induced by NAD deficiency alone or HFD, whereas adenovirus-mediated SIRT1 overexpression only partially rescued these phenotypes [7]. This study suggests that other downstream of NAD also participates in Nampt mediated effects on liver metabolism. Further research is needed to demonstrate whether impaired lipid metabolism caused by hepatic Nampt deficiency is associated with Nampt/NAD/SIRT1 and other downstream of NAD.

The liver is the central organ that controls lipids homeostasis and precisely regulates biochemical, signaling, and cellular pathways [32]. Hepatocytes are the main liver parenchymal cells, which control hepatic biochemical and metabolic functions in the liver, including TG and TC metabolism [33,34]. Several studies have linked liver and metabolic syndrome, especially abdominal obesity, insulin resistance, and increased serum TG and LDL-C [35]. In the present study, we observed abnormal liver metabolic function in HC-Nampt^-/-^ mice fed with HFD, with increased serum TG and TC and accumulated neutral lipids, such as TG, in the liver. TG molecules represent the major form of storage and transport fatty acids within cells and serum. The liver is the main site where TG is synthesized from fatty acids and secreted into the circulatory system [36]. The liver only stores small amounts of TG under normal circumstances, but in metabolic disorders caused by enhancive lipids intake, TG metabolism and transport are altered, which leads to an accumulation of TG within hepatocytes, causing fatty liver [37,38]. Oil red O is a fat-soluble dye that can specifically stain neutral fats, such as TG, in tissues. In this study, the increased area of oil red O staining in the HC-Nampt^-/-^ mice was directly associated with the accumulation of TG under HFD. Our results further support the idea that there is a correlation between fatty liver and dyslipidemia. Both serum and liver TG levels may be elevated in the presence of metabolic-associated fatty liver disease and liver fibrosis [39,40]. Animal studies have shown that the accumulation of cholesterol in the liver plays a crucial role in the development of fatty liver [41]. Excess cholesterol in the liver results in more cholesterol to be retained in the circulatory system and liver by decrease in the conversion of liver cholesterol to bile acids [42,43]. In line with those studies, we previously found that, in systemic NAD deficiency mice, the liver TG and cholesterol contents were increased, neutral fat accumulation and fibrotic lesions appeared in liver and lipid homeostasis was impaired [7]. In this study, we further clarified that TC and TG increased under HFD in the absence of hepatic Nampt. These results suggest that hepatic Nampt can maintain liver lipids metabolism, then maintain the TG and TC balance between liver and serum, when lipids intake increases. Consistent with this, an increase in liver Nampt exhibits significantly elevated intracellular NAD levels and decreased ethanol-induced TG accumulation [44].

The adipose tissue is also a key factor in lipid metabolism. Nampt is highly expressed in adipose tissue, and the adipokine name visfatin is mainly for Nampt secreted from the adipose tissue. In the present study, hepatocyte-specific Nampt knockout mice exhibited Nampt deficiency only in the liver, rather than in adipose and other tissues (Figure 1). Thus, we did not check the adipose tissue. However, the effect of the crosstalk between liver and adipose tissue on lipid metabolism produced by hepatic Nampt deficiency needs to be further studied in the future.

This study only used the HFD-fed model. In our previous study, hepatic Nampt-NAD axis was impaired in aged mice and humans, and middle-age mice with systemic Nampt mutant overexpression displayed systemic NAD reduction and had moderate fatty liver phenotypes under NCD, which was deteriorated further under HFD challenge [7]. Thus, the present findings perhaps also occur in the ageing-related dyslipidemia and fatty liver. The hepatopathy induced by aging in HC-Nampt^-/-^ mice will be further investigated in the future.

In summary, at baseline state, hepatic Nampt deficiency has no effect on serum lipids levels and liver weight. When lipids intake increases, the deficiency of hepatic Nampt aggravates dyslipidemia and fat accumulation in the liver. Therefore, hepatic Nampt can be a protective target against dyslipidemia and fatty liver. As with fatty liver, atherosclerosis is also an ageing-associated disease. The incidence of these two kinds of diseases increases with age. Considering that systemic upregulation of Nampt-NAD axis may have the susceptibility of atherosclerosis, our present study indicates that hepatocyte Nampt is a better target than systemic Nampt for prevention and treatment of dyslipidemia and fatty liver.

## Figures and Tables

**Table 1:** Primer sequences used to amplify mice target genes.

Mouse Genes	Accession NCBI	Forward Primer 5′–3′	Reverse Primer 5′–3′
Nampt	59,027	GCAGAAGCCGAGTTCAACATC	TTTTCACGGCATTCAAAGTAGGA
GAPDH	14,433	AGGTCGGTGTGAACGGATTTG	TGTAGACCATGTAGTTGAGGTCA

## Data Availability

All data from the study are given in the manuscript.

## Supplementary Materials

The following supporting information can be downloaded at: https://www.mdpi.com/article/10.3390/cells12040568/s1, Figure S1: The representative pictures of the middle lobes of liver from WT and HC-Namp^t-/-^ mice under NCD and HFD for 10 weeks. Figure S2: The representative pictures of the liver from WT and HC-Nampt^-/-^ mice under NCD and HFD for 8 days. Table S1: Body weight, liver weight and liver weight index (the ratio of the liver weight to the body weight) in WT and HC-Nampt^-/-^ mice under NCD and HFD for 8 days. Refs [45,46] are mentioned in supplementary materials.
