# Supplementary material for: Hepatic Nampt Deficiency Aggravates Dyslipidemia and Fatty Liver in High Fat Diet Fed Mice
Source: Cells. 2023 Feb 10;12(4):568. doi: 10.3390/cells12040568 (PMC9954480; doi:10.3390/cells12040568)
Supplement: Supplementary file 1 [file cells-12-00568-s001.zip › cells-2098614-supplementary.pdf]

Supplementary Results

The middle lobes of the mice liver stored at −80 °C were thawed and photographed. As shown in Figure S1, liver tissues from wild-type (WT) and hepatocyte-specific Nampt knockout (HC-Nampt$^{-/-}$) mice under normal chow diet (NCD) displayed similar color and texture. However, under high fat diet (HFD) for 10 weeks, liver tissues were obviously different from those under NCD, and liver tissue in HC-Nampt$^{-/-}$ group appeared darker in color and harder in texture when compared with that in WT group.

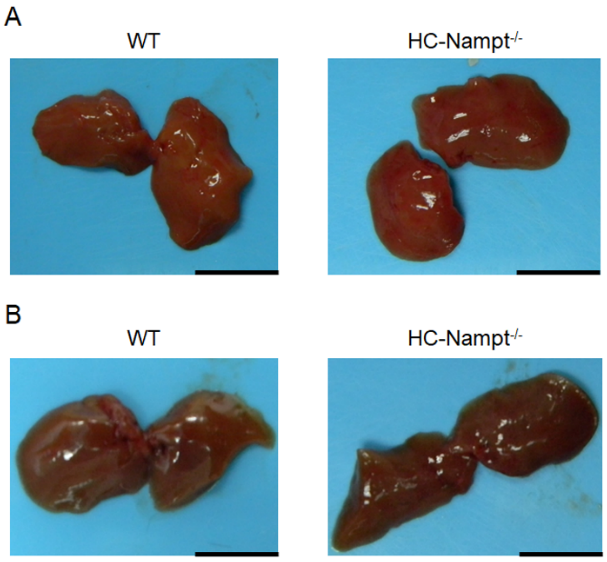

Figure S1. The representative pictures of the middle lobes of liver from WT and HC-Nampt$^{-/-}$ mice under NCD (**A**) and HFD for 10 weeks (**B**). The tissues stored at −80 °C were thawed for photography. Scale bar = 1 cm.

Additional WT and HC-Nampt$^{-/-}$ mice were used. Some mice were fed HFD for 8 days and then sacrificed by an overdose of pentobarbital sodium (100 mg·kg^{-1}, i.p.) for photography. Under NCD, WT and HC-Nampt$^{-/-}$ mice displayed similar liver weight index (3.96% for the WT mouse and 4.00% for the HC-Nampt$^{-/-}$ mouse, Table S1), as well as similar liver color and texture (Figure S2). Under HFD for 8 days, liver weight index in HC-Nampt$^{-/-}$ group (5.11% and 5.08% for two HC-Nampt$^{-/-}$ mice) appeared larger than that in WT group (4.53%, 4.38% and 4.04% for three WT mice) (Table S1). Due to very limited number of the mice, statistical analysis for these data cannot be performed. Nevertheless, an increase trend in liver weight index was observed by HFD (HFD vs NCD), as well as by hepatic Nampt deficiency under HFD (HC-Nampt$^{-/-}$ vs WT under HFD) (Table S1). Further, under HFD some white lipid spots on the surface of liver tissue, as described in the previous studies [1,2], were observed in two Nampt$^{-/-}$ mice, but not in three WT mice (Figure S2).

In summary, there were no changes in liver gross phenotypes between WT and HC-Nampt$^{-/-}$ mice at baseline state. However, there were alterations in liver gross phenotypes between WT and HC-Nampt$^{-/-}$ mice under HFD challenge. The limitations for these gross detection experiments are: (1) Iced liver tissues stored at −80 °C were used for Figure S1. The very low temperature may affect the liver color and other gross phenotypes; (2) In Figure S2, although the liver tissues were fresh, the time duration for HFD was only 8 days. This short-term HFD for 8 days is different from the long-term HFD for 10 weeks presented in the main text of the study.

Table S1. Body weight, liver weight and liver weight index (the ratio of the liver weight to the body weight) in WT and HC-Nampt$^{-/-}$ mice under NCD and HFD for 8 days.

Genotype	Diet	Body weight (g)	Liver weight (g)	Liver weight/body weight (%)
WT	NCD	27.8	1.10	3.96
HC-Nampt$^{-/-}$	NCD	30.2	1.21	4.00
WT	HFD	27.6	1.25	4.53
WT	HFD	23.5	1.03	4.38
WT	HFD	27.7	1.12	4.04
HC-Nampt$^{-/-}$	HFD	26.2	1.34	5.11
HC-Nampt$^{-/-}$	HFD	26.0	1.32	5.08

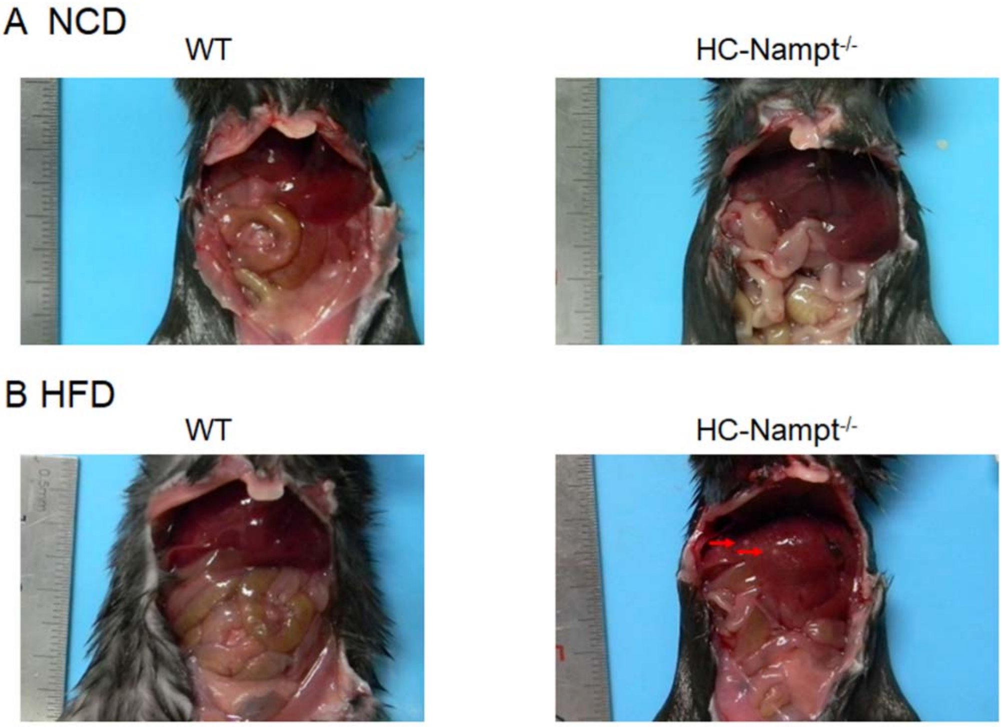

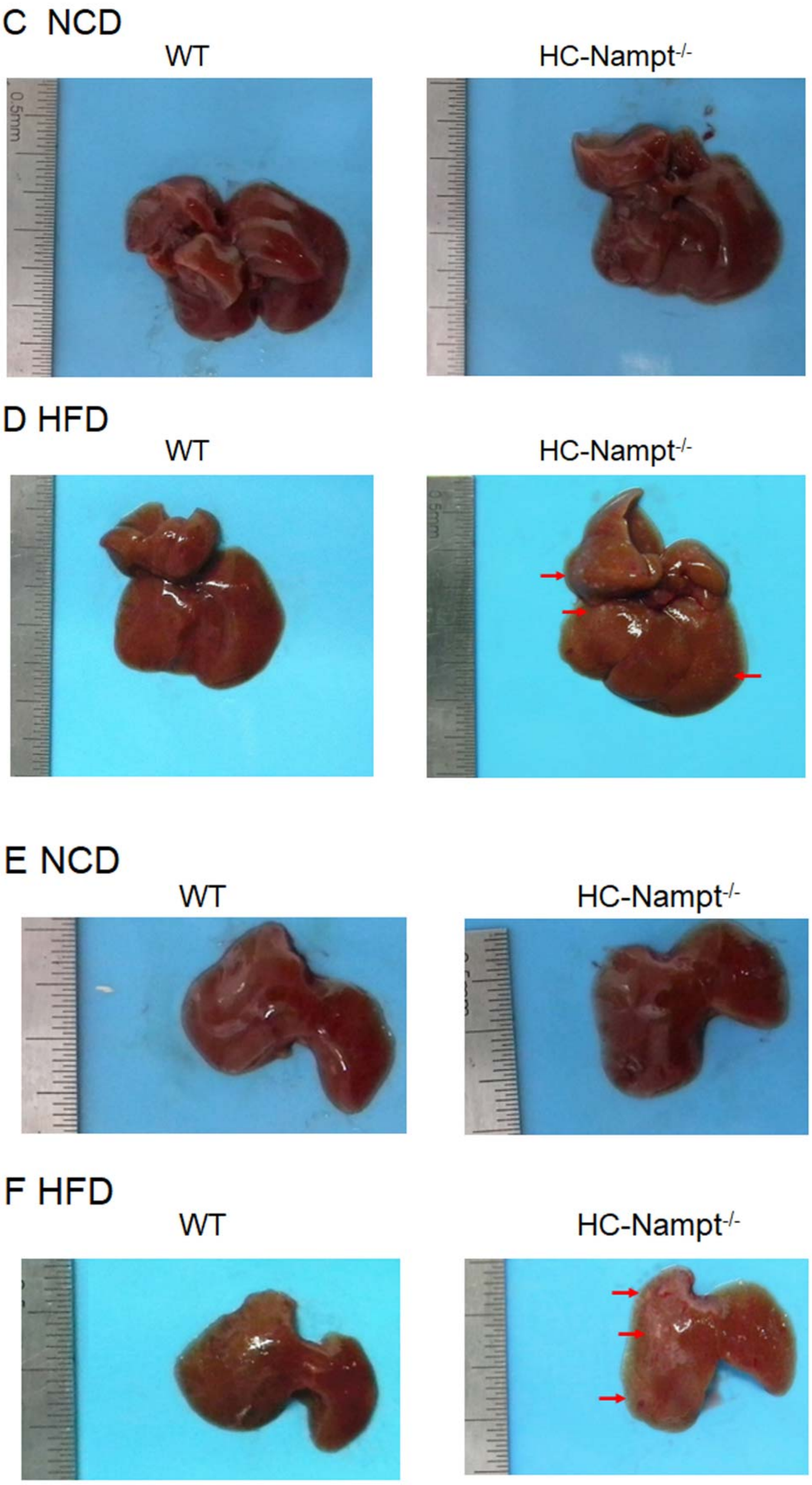

Figure S2. The representative pictures of the liver from WT and HC-Nampt$^{-/-}$ mice under NCD and HFD for 8 days. (**A** and **B**) The representative pictures of the whole liver in the animal abdominal cavity from WT and HC-Nampt$^{-/-}$ mice under NCD (**A**) and HFD for 8 days (**B**). Scale bar = 4 cm. (**C** and **D**) The representative pictures of the whole isolated liver from WT and HC-Nampt$^{-/-}$ mice under NCD (**C**) and HFD for 8 days (**D**). Scale bar = 4 cm. (**E** and **F**) The representative pictures of middle lobe of liver from WT and HC-Nampt$^{-/-}$ mice under NCD (**E**) and HFD for 8 days (**F**). Scale bar = 2 cm.

References

1. Brown, G.T.; Kleiner, D.E. Histopathology of nonalcoholic fatty liver disease and nonalcoholic steatohepatitis. *Metabolism: clinical and experimental* **2016**, *65*, 1080–1086, doi:10.1016/j.metabol.2015.11.008.
2. Wree, A.; Schlattjan, M.; Bechmann, L.P.; Claudel, T.; Sowa, J.P.; Stojakovic, T.; Scharnagl, H.; Köfeler, H.; Baba, H.A.; Gerken, G.; et al. Adipocyte cell size, free fatty acids and apolipoproteins are associated with non-alcoholic liver injury progression in severely obese patients. *Metabolism: clinical and experimental* **2014**, *63*, 1542–1552, doi:10.1016/j.metabol.2014.09.001.
